# The Kickstart Walk Assist System for improving balance and walking function in stroke survivors: a feasibility study

**DOI:** 10.1186/s12984-020-00795-y

**Published:** 2021-02-24

**Authors:** Jiajia Yao, Takashi Sado, Wenli Wang, Jiawen Gao, Yichao Zhao, Qi Qi, Mukul Mukherjee

**Affiliations:** 1 Shanghai YangZhi Rehabilitation Hospital (Shanghai Sunshine Rehabilitation Center), Tongji University School of Medicine, Shanghai, China; 2 Department of Biomechanics, University of Nebraska at Omaha, Omaha, NE USA

**Keywords:** Gait, Exoskeleton, Exotendon, Wearable, Robotics, Rehabilitation, Walking

## Abstract

**Background:**

Compared with traditional physical therapy for stroke patients, lower extremity exoskeletons can provide patients with greater endurance and more repeatable and controllable training, which can reduce the therapeutic burden of the therapist. However, most exoskeletons are expensive, heavy or require active power to be operated. Therefore, a lighter, easy to wear, easy to operate, low-cost technology for stroke rehabilitation would be a welcome opportunity for stroke survivors, caregivers and clinicians. One such device is the Kickstart Walk Assist system and the purpose of this study was to determine feasibility of using this unpowered exoskeleton device in a sample of stroke survivors.

**Methods:**

Thirty stroke survivors were enrolled in the study and experienced walking with the Kickstart exoskeleton device that provided spring-loaded assistance during gait. After 5 days of wearing the exoskeleton, participants were evaluated in the two states of wearing and not wearing the exoskeleton. Outcome measures included: (a) spatio-temporal gait measures, (b) balance measures and (c) exoskeleton-use feedback questionnaire.

**Results:**

In comparison to not wearing the device, when participants wore the Kickstart walking system, weight bearing asymmetry was reduced. The time spent on the 10-m walk test was also reduced, but there was no difference in the timed-up-and-go test (TUGT). Gait analysis data showed reduction in step time and double support time. Stroke survivors were positive about the Kickstart walking system’s ability to improve their balance, speed and gait. In addition, their confidence level and willingness to use the device was also positive.

**Conclusions:**

These findings show the feasibility of using the Kickstart walking system for improving walking performance in stroke survivors. Our future goal is to perform a longer duration study with more comprehensive pre- and post-testing in a larger sample of stroke survivors.

*Trial registration* Chinese Clinical Trial Registry, ChiCTR2000032665. Registered 5 May 2020—Retrospectively registered, http://www.chictr.org.cn/showproj.aspx?proj=53288

## Background

Nearly one-third of strokes occur in people over the age of 65, and most stroke survivors have associated ambulation problems [1, 2]. In this population, the reduction in muscle mass and muscle strength frequently reduces their daily activities, confines them to bed, and reduces the ability to move, thus accelerating the degradation of the neuromuscular system. Stroke can lead to major impairments related to functional mobility [3–5] that consequently impacts independence and reduces the quality of life. Conventional gait therapy for stroke survivors, that is provided by therapists, can improve gait speed and endurance [6], especially when performed in the sub-acute stage [7]. However, it is demanding and exhausting for therapists and outcomes depend on the skill of the therapist which may vary a lot depending on experience and expertise. Devices that reduce this burden like the body weight support system or robot-assisted gait training devices like the Lokomat have other issues such as being too expensive and bulky, and may require superior technical skills to operate and therefore may not suitable for wide usage [8]. Therefore, in recent years, light and easy-to-operate exoskeletons have become popular which can help stroke survivors who are unable to stand independently to regain their ability to stand and walk [9].

Exoskeletons have been in development since at least the 1890′s [10]. In the past several decades, many universities, research institutions and companies have made great progress in developing exoskeleton-assisted rehabilitation devices [11, 12]. Based on power source types, exoskeletons can be categorized as active (powered by the external sources) or passive (self-powered through elastic components) [13–15]. Currently, several lower extremity exoskeletons are in the market that can assist with gait training in stroke survivors, including treadmill-based Lokomat [16], LokoHelp [17] and ReoAmbulator [18], and wearable systems such as Ekso GT [19], HAL-5 [20] and ReWalk [21]. They are mainly used for elderly people or patients who have lost walking ability due to stroke or spinal cord injury, so that they can walk, sit up, and climb stairs, thus reducing the burden on the caregiver and improving the quality of life of the patients [22].

Exoskeletal devices target characteristic deficiencies observed after a stroke—insufficient forward propulsion, reduced range of motion, hyper-reflexia which lead to compensatory strategies such as hip hiking, circumductory gait and elevated metabolic cost [23–27]. Compared with traditional physical therapy, lower extremity exoskeletons can provide patients with more repeatable and controllable training, which can reduce the treatment burden of the therapist, so that the therapist can pay attention to other aspects of the patient’s treatment [28].

Based on a Cochrane review of electromechanical and robotic-assisted training for walking after stroke [29], an analysis of 36 different research studies that involved over 1400 participants, it was shown that the use of such devices in combination with physical therapy can improve walking after stroke. These devices were shown to be safe and acceptable to most participants. It was noted that the improvement was greatest for those who were not ambulatory. Additionally, best results were obtained for treatment in the acute/sub-acute phase within 3 months of the stroke episode. It is important to note that a reason for the Cochrane review of this field was to determine the justification for large equipment and human resource costs that are needed to implement electromechanical-assisted gait devices.

Although in the past 20 years, research and development of robotic exoskeletons has grown rapidly, and many robotic-assisted systems have been successfully used in scientific research and clinical applications, the adoption rate remains very low. According to a survey of 1326 rehabilitation therapists in the US, about 2% of them used some robot-assisted devices for upper and lower movement rehabilitation [30]. Primary barriers to adoption include the lack of scientific evidence of effectiveness, high economic costs and low user-friendliness, which greatly limit clinical usage of robotic exoskeletons [31, 32]. While active devices, like Lokomat, provide several functional benefits to a variety of patient populations, these are only available to well-funded clinical facilities and research settings. Moreover, it is unrealistic for patients to use an active device by themselves because of its size, weight, cost, and complexity of use. Therefore, there is a significant need for lighter-weight, easy to wear, user-friendly, and low-cost technologies for walking training in stroke survivors.

**Fig. 1:**
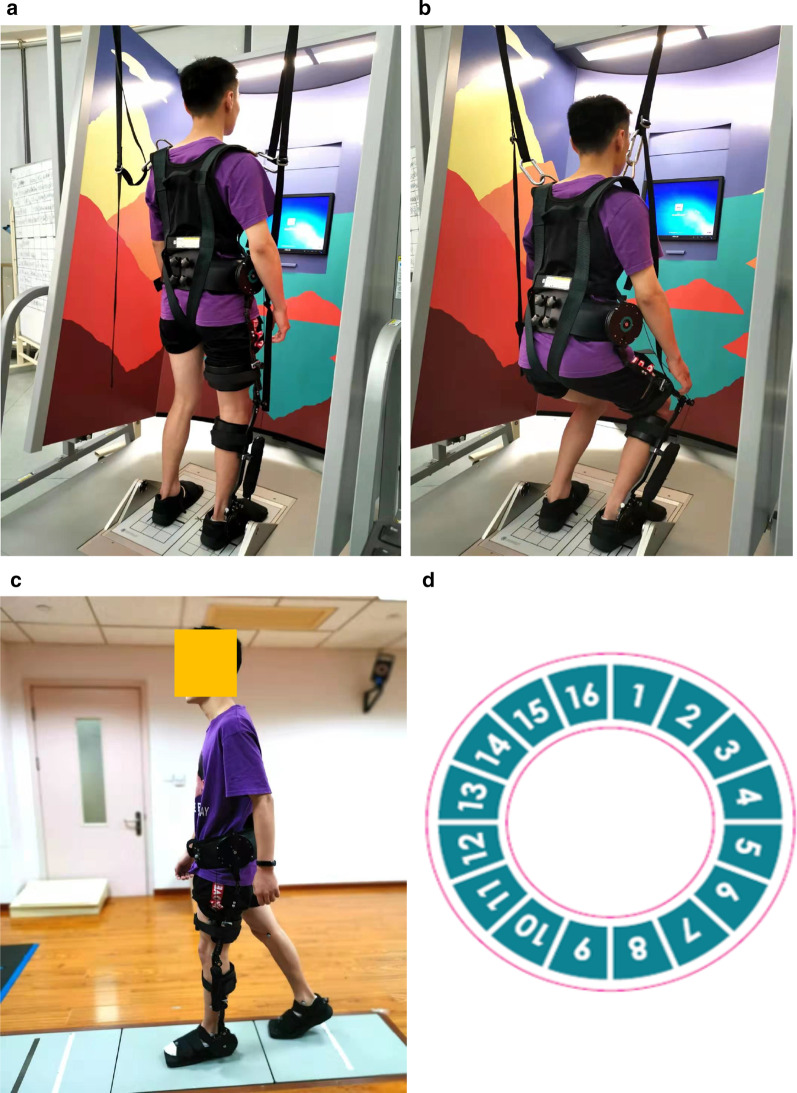
The figure shows the setup for the weight bearing squat test in the **a** upright position and **b** The squatting position with the exoskeleton device attached unilaterally. **c** Shows a non-study participant walking with exoskeletal assistance on a set of force platforms in the gait lab that has motion capture cameras on the walls. **d** Shows the dial sticker that was attached to the hip piece to note the movement of the ratchet for tightening the exotendon cable

The Kickstart^®^ Walk Assist system is such a rehabilitation device that consists of a belt, an external support structure and an Exotendon (Fig. 1). The effect of the Exotendon is similar to an artificial tendon, which stores energy during the stance phase and releases it during the swing phase of the gait cycle. The Exotendon mechanism is inspired by the anatomical features of the hind limbs of the horse: in the hind limbs of the horse, several long tendons span multiple joints, and during the stance phase, the tendons stretch and store energy, and this stored energy is then used to initiate gait swing and consequently, reduce muscle exertion [33, 34]. Compared to other robotic lower extremity exoskeleton systems, the Kickstart walking system is lighter in weight, easier to wear and take off, and is inexpensive. In a series of case studies (2 stroke survivors and one spinal cord injury patient), it was shown that the Kickstart walking system could increase wearers' walking speed and endurance [35]. Unlike more tightly controlled exoskeletal systems like the Lokomat, the lightweight, spring-loaded Kickstart walking system could allow easier interaction with the environment that would be more explorative.

The purpose of this study was to determine the feasibility of using the Kickstart walking system in a sample of stroke survivors who were in the subacute and chronic stages of the disease and attending an inpatient rehabilitation center. Study participants were tested for several measures with/without the device after experiencing walking with the exoskeleton over a period of 5 days. Outcome measures included: (a) gait measures, (b) balance measures and (c) exoskeleton-use feedback questionnaire. Results from this study will help us to explore if the technology can offer a new option for encouraging the recovery of walking ability of stroke patients, optimizing the rehabilitation treatment strategy, and providing some reference for subsequent related research.

## Methods

### Experimental subjects

In this study, a sample of 30 stroke survivors, were recruited from the Shanghai Yangzhi Rehabilitation Hospital (Shanghai Sunshine Rehabilitation Center), Tongji University School of Medicine (Additional file 1: CONSORT flow diagram). Each participant was required to sign an informed consent form approved by the hospital’s review board. Volunteers participating in the experiment were those admitted to the inpatient rehabilitation center between September and December of 2018. Study participants met the following inclusion criteria: (1) diagnostic criteria for stroke by the Fourth Chinese National Cerebrovascular Disease Conference, modified from the standard WHO definition of stroke; (2) confirmation by cranial CT/MRI; (3) diagnosis of primary subcortical ischemic stroke with a disease duration greater than 1 month; (4) Ability to walk alone > 20 m with or without a walking aid. The exclusion criteria were the following: (1) a history of severe arrhythmia; (2) peripheral nerve injury; (3) uncontrolled hypertension; (4) severe orthopedic conditions; (5) chronic pain; and (6) severe cognitive impairment.

### Experimental process

Each study participant was assigned a trained physical therapist who fitted the patient with the passive exoskeleton device and assessed their walking and balancing ability with or without the device. Prior to the experiment, each study participant attended at least one training session of duration 20 min per day for 5 days with the exoskeleton device while they carried out their routine rehabilitation activities. After the 5-day familiarization period, each participant was assessed in the following tasks with and without an exoskeleton: (1) the 10-m walking test or 10-MWT; (2) the *Timed Up and Go* or TUG test; (3) the *Weight Bearing/Squat* Test; (4) Gait analysis during over ground walking, and (5) Feedback questionnaire.

### The exoskeleton device

The passive gait assistance device used in this study was the Kickstart^®^ Walk Assist system (Real Star Rehabilitation, Shanghai, China). This device is a passive exoskeleton device with an Exotendon that runs parallel to the lateral side of the leg and goes through pulleys over the hip, knee and ankle joints. Before each training session, the exoskeleton device was attached to the more affected limb of the subject. After the device was firmly attached, tightness of the exotendon was adjusted using a ratchet attached to the disk located on the side of waist belt until the subject felt the assist and could clear his/her foot off the ground. The number of ratchet clicks was noted using a sticker attached to the disk (Fig. 1d).

### Evaluation methods

#### Walking efficiency: this was evaluated using the 10-MWT and the TUG Test

1. The 10-MWT: in this test, the set distance between the starting and the end point is 10 m. One meter is added at either end of the set distance to allow the participant to accelerate and decelerate. The experimenter started timing with a stopwatch when the subject initiated walking from the starting line and stopped timing as the subject reached the finish line. Three trials were performed each with and without the exoskeleton device and the results were averaged and compared.
2. The TUG Test: the participant started in a seated position and upon hearing a "start" command, stood up from the chair, walked 3 m forward at their own comfortable walking pace, turned around over the thick line or mark, walked back to the chair and sat down. No physical help was given during the test. The experimenter recorded the time (in seconds) it took the participant to complete the test. Three trials were performed each with and without the exoskeleton device and the results were averaged and compared.

#### Balance performance

This was evaluated using the *Weight Bearing/Squat* test of the NeuroCom Balance Master (Neurocom International, Clackamas, OR). Relative weight bearing on each limb of each patient were measured without and with the exoskeleton device. Before testing, the patient was fitted with a harness for safety and stood on the force plate to align his/her center of gravity with the center of the screen (Fig. 1a, b). In this test, the percentage of body weight borne on each limb is calculated at different knee flexion angles. Each participant was asked to flex his/her knee joints by 0°, 30°, 60°, and 90° and the percentage of weight bearing at each flexion angle was used to calculate differences in weight bearing between the limbs (unimpaired–impaired). This was averaged across the four flexion angles. The higher the value, more asymmetrical is the weight bearing between the legs.

#### Gait recording and analysis of the overground walking trials

Each participant performed a 6-m overground walking trial each with and without the exoskeleton. Each participant’s gait was tracked with an 8-camera 3D Motion capture System (Vicon Motion Systems Ltd. UK) at a sampling frequency of 100 Hz using 21 retro-reflective markers. These markers were placed at specific anatomical landmarks. A lower body marker set was used (the plug-in-gait lower body model) that included the anterior and posterior superior iliac spines, sacrum, lateral and medial markers at the knee and ankle, tibia, thigh, heel and toe. Several kinematic parameters were analyzed using the Vicon Nexus software (version 1.8.5; Vicon Motion Systems Ltd. UK). These were the following: cadence, walking speed, double support time (DST), limp index, step length, step time, and step width. These were calculated in the following way:

1. *Cadence*: the average number of steps/minute.
2. *Walking speed of the specific foot:* separately calculated for the impaired and non-impaired foot from impaired and non-impaired stride length and stride time and then averaged.
3. *Step length, stride length, step time, stride time, DST, single support time and step width* follow standard definition. For example, step length is the distance measured from foot contact of the more impaired foot to the foot contact by the opposite foot.
4. *Limp Index*: this is the ratio of the total support time (sum of single and double) of the more impaired foot divided by the total support time of the opposite foot. For symmetric walk, the limp index is exactly 1. For the impaired foot, limp index is less than 1, while the index for the opposite foot greater than 1.

### Feedback questionnaire

Study participants filled out a 1–5 Likert-scale based questionnaire that had 8 items. These were the subjective perceptions of: gait improvement, speed improvement, stability improvement, ease of wearing the device, level of comfort, confidence level, willingness to use and peer recommendation.

### Statistical methods

Statistical analysis was done using SPSS 22.0 software. This experiment was a single-sample experimental research design with each subject performing balance and gait tasks with and without the exoskeletal device after 5-days of being familiarized with the device. Paired t-tests were done to compare the dependent variables for the same sample group with and without the exoskeleton device. A value of *p* < 0.05 was considered statistically significant.

## Results

### Demographics of the study participants

The demographics of the study participants is provided in Table 1. The average age of the subjects was (52.57 ± 2.28 years) old, and the average onset time was (7.27 ± 1.05 months). Among the subjects, 27 subjects were male and 3 were female. About half of the study participants were impaired on the right side (n = 16/30). Twenty of the subjects had cerebral infarction and 10 suffered from hemorrhagic stroke. About half of all subjects (n = 16) were able to walk independently while the others used an assistive device. No adverse events occurred for any of the participants in the study.

**Table 1:** Demographics of the study participants

Subject #	Stroke diagnosis	Age (years)	Sex	Side impaired	Disease onset (months)	Assistive device	Exotendon Scale (0-N)
1	Hemorrhagic	53	Male	Right	5	None	0
2	Ischemic	60	Male	Left	11	None	0
3	Hemorrhagic	65	Female	Left	9	Walker	0
4	Ischemic	53	Male	Right	4	None	0
5	Ischemic	57	Male	Left	7	None	0
6	Hemorrhagic	38	Male	Left	28	Crutch	0
7	Ischemic	50	Male	Right	5	None	0
8	Ischemic	62	Male	Right	2	None	0
9	Hemorrhagic	38	Male	Left	6	Walker	0
10	Ischemic	51	Male	Right	12	None	0
11	Hemorrhagic	27	Female	Right	3	Walker	5
12	Ischemic	51	Male	Right	3	Walker	5–7
13	Hemorrhagic	52	Male	Right	3	None	4–6
14	Hemorrhagic	32	Male	Right	19	None	5–6
15	Ischemic	61	Male	Left	10	Crutch	6
16	Ischemic	48	Male	Left	1	None	7
17	Ischemic	55	Male	Left	2	None	6
18	Ischemic	55	Male	Right	3	None	7
19	Ischemic	46	Male	Right	6	None	5
20	Ischemic	63	Male	Left	4	Walker	7
21	Ischemic	60	Male	Right	13	Walker	6
22	Hemorrhagic	76	Female	Left	7	Crutch	5
23	Ischemic	79	Male	Right	6	Walker	7
24	Hemorrhagic	58	Male	Left	15	Crutch	6
25	Ischemic	26	Male	Left	4	None	7
26	Ischemic	42	Male	Left	6	None	5
27	Ischemic	68	Male	Left	4	Crutch	6
28	Ischemic	52	Male	Right	11	Walker	5
29	Ischemic	52	Male	Right	5	None	5
30	Hemorrhagic	47	Male	Right	4	Walker	5
N = 30	Hemorrhagic = 10 Ischemic = 20	52.57 ± 2.28	Male = 27 Female = 3	Right = 16 Left = 14	7.27 ± 1.05	Device = 14 None = 16	Range 4–7

The last row provides the total number of subjects as well as the break-up of hemorrhagic/ischemic, male/female, right/left affected, and the use of assistive/no devices used. In addition, it also provides the mean ± standard deviations for the age, disease duration and also the range of Exotendon stiffness scale

### Balance and walking function

#### Walking efficiency

**Fig. 2:**
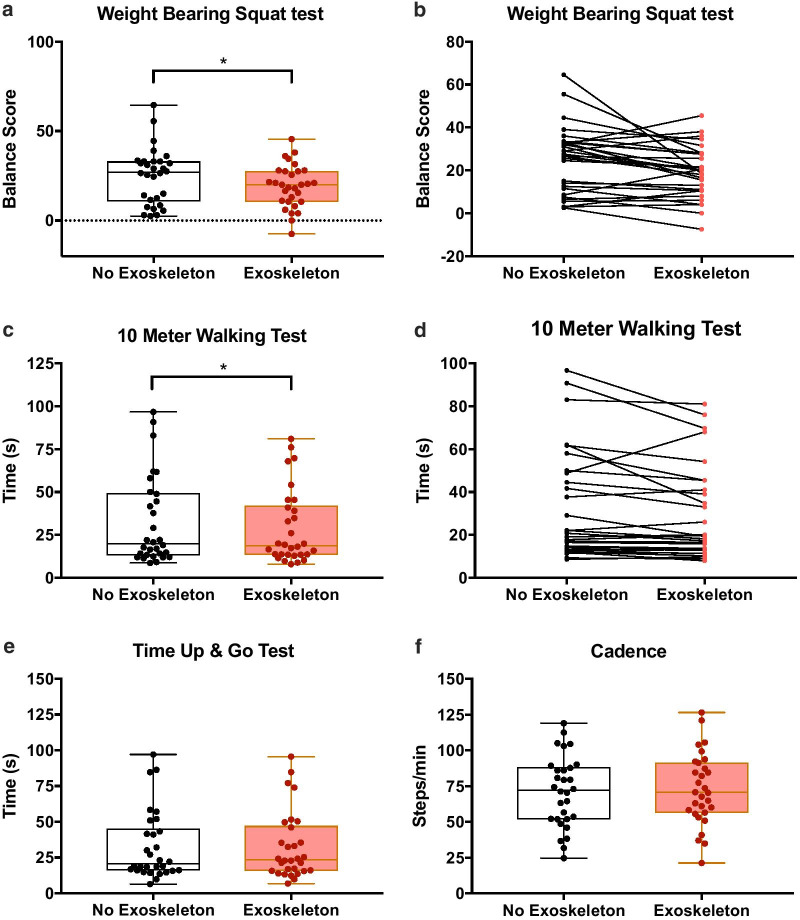
Box and whisker plots of 1–99 percentile (whiskers) and 25–50–75 percentile (box) for the **a** weight bearing squat test, **c** 10-m walking test, **e** Timed up and go test and **f** cadence. Individual scores for stroke participants with and without exoskeleton are provided for **b** weight bearing squat test, and **d** 10-m walking test. *p < 0.05

Figure 2c and e show the results of the 10MWT and the TUG Test respectively. For the 10MWT (Figure 2c and d), walking for 5 days with the exoskeleton device resulted in a significantly shorter time spent (*p* = 0.036, t_1,29_ = 2.201) on average to cover 10 m while wearing the device (27.52 ± 22.14 s) than without (30.81 ± 26.09 s). For the TUG test, there was no significant difference between the two conditions (Fig. 2e; *p* = 0.991, t_1,29_ = 0.011).

#### Balance function

Figure 2a and b show the results of the *Weight Bearing/Squat* test. Walking for 5 days with the exoskeleton device resulted in a significant reduction in the asymmetrical weight bearing between the legs. This asymmetry was significantly lower (*p* = 0.011, t_1,29_ = 2.733) while wearing the device (19.13 ± 12.01%) than without (24.83 ± 15.5%).

#### Spatio-temporal gait measures during overground walking trials

**Fig. 3:**
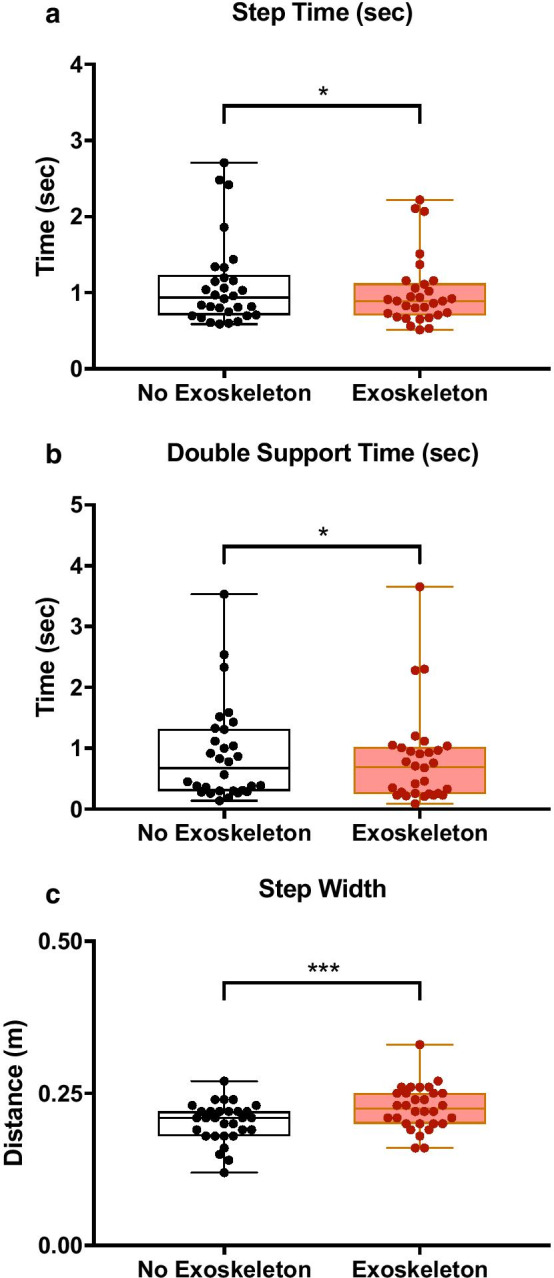
Box and whisker plots of 1–99 percentile (whiskers) and 25–50–75 percentile (box) for the **a** step time, **b** double support time, and **c** step width. *p < 0.05 and ***p < 0.001

The results of gait analysis demonstrated the acute effects of walking with an exoskeleton device for 5 days in our sample of stroke survivors. For step length (Table 2), walking for 5 days with the exoskeleton device did not result in a significant change for step initiated from the impaired (*p* = 0.857, t_1,29_ = 0.181) side. However, step time changed (Fig. 3a), resulting in a significantly reduced duration (*p* = 0.019, t_1,29_ = 2.472) when wearing the device (1.001 ± 0.448 s) than not (1.104 ± 0.566 s). DST (Fig. 3b), also showed a significantly reduced duration (*p* = 0.0205, t_1,29_ = 2.452) when wearing the device (0.805 ± 0.768 s) than not (0.900 ± 0.796 s). Finally, step width (Fig. 3c) was significantly increased (*p* = 0.001, t_1,29_ = 3.665) when wearing the device (0.226 ± 0.036 m) than not (0.203 ± 0.032 m). Other gait variables (Table 2) were not significantly impacted by exoskeleton assistance. These included walking speed (*p* = 0.267, t_1,29_ = 1.131), cadence (*p* = 0.343, t_1,29_ = 0.964; Fig. 2f) and limp index (*p* = 0.453, t_1,29_ = 0.761).

**Table 2:** Descriptives of the dependent variables along with the results of paired comparisons

Variable	Condition	Mean ± SD	*tI*	*p*
10MWT (s)	No exoskeleton	30.81 ± 26.09	2.201	**0.036**
	Exoskeleton	27.52 ± 22.14		
TUG (s)	No exoskeleton	32.58 ± 24.08	0.011	0.991
	Exoskeleton	32.60 ± 23.61		
WBS (%)	No exoskeleton	24.83 ± 15.5	2.733	**0.011**
	Exoskeleton	19.13 ± 12.01		
Cadence (steps/min)	No exoskeleton	71.09 ± 24.74	0.343	0.964
	Exoskeleton	73.01 ± 25.06		
Walking speed (m/s)	No exoskeleton	0.498 ± 0.314	1.131	0.267
	Exoskeleton	0.519 ± 0.346		
Step length (m)	No exoskeleton	0.403 ± 0.182	0.181	0.857
	Exoskeleton	0.400 ± 0.182		
Step time (s)	No exoskeleton	1.104 ± 0.566	2.472	**0.019**
	Exoskeleton	1.001 ± 0.448		
DST (s)	No exoskeleton	0.900 ± 0.796	2.452	**0.021**
	Exoskeleton	0.805 ± 0.768		
Step width (m)	No exoskeleton	0.203 ± 0.032	3.665	**0.001**
	Exoskeleton	0.226 ± 0.036		
Limp Index	No exoskeleton	0.878 ± 0.101	0.761	0.453
	Exoskeleton	0.891 ± 0.125		

The mean ± standard deviations for each condition are provided for all the dependent variables. In addition, the t-statistic for the paired comparisons along with their significance is also provided

10MWT is the *10-m walk test*

TUG is the *timed up and go test*

WBS is the *Weight Bearing/Squat* test

DST is the double support time

Limp index is unitless because it is a ratio of double support times

### Exoskeleton-use feedback questionnaire

Results were not recorded from two study participants. Results from the remaining 28 subjects showed that on a scale of 1 (least positive perception) to 5 (most positive perception), stroke survivors on average, perceived their exoskeleton experience as more positive (> 3) for stability improvement (3.86 ± 0.76), speed improvement (3.64 ± 0.87) and gait improvement (3.57 ± 0.74). In addition, their confidence level (3.59 ± 0.80) and willingness to use (3.14 ± 1.04) was also more positive which was reflected in viewing peer recommendation (3.54 ± 1.10) more positively. The study participants were less positive (< 3) about the ease of wearing the device (2.71 ± 0.94), and their level of comfort (2.68 ± 0.90).

### Additional analysis of interest

We analyzed our outcome measures for normality and found that our variables of interest were not normally distributed. These included Balance, 10MWT, TUG test, step time and DST. Only step width was found to show normal distribution. We performed non-parametric analysis with all these variables of interest. Non-parametric analysis using the related-samples Wilcoxon signed rank test revealed significant differences between wearing and not wearing the exoskeleton device for balance (p = 0.007), 10MWT (p = 0.021), step time (p = 0.032) step width (p = 0.001) and DST (p = 0.019). No differences for walking speed (p = 0.726) and the TUG test (p = 0.797) were determined. These results mirrored our findings with the parametric analysis. Since acute and chronic (> 6 months) stages of stroke could possibly skew the results, we did further analysis with 15 subjects in each of the acute and chronic stages. We found no significant differences between the two stages for our variables of interest. We also analyzed the minimum detectable change (MDC) values for the variables of interest specifically those that were statistically significant. For example, the MDC for the 10MWT for our sample was 10.06 that was well above the statistically significant difference between the two exoskeleton conditions. However, in this short-term exoskeleton study, our objectives were more towards feasibility of wearing and training with the device in a sample of stroke survivors rather than determining significant treatment outcomes. We are using our results from this study to perform a currently ongoing longer duration study where treatment outcomes are our main objective. In that study, we will look at MDC and MCID more closely.

## Discussion

This study was performed to determine the feasibility of using the Kickstart^®^ Walk Assist system in a sample of stroke survivors. The study was specifically done to determine if there were any balance, gait or comfort issues encountered when using the device. Study participants were tested for several measures with/without the device after experiencing walking with the exoskeleton over a period of 5 days. Study outcome measures demonstrated that using the device for longer periods would be feasible for stroke survivors.

### Balance measures

Previous studies have shown that weight-bearing tends to better represent the body's balance function [36, 37]. We used the *Weight Bearing/Squat* test of the NeuroCom Balance Master to record the relative weight bearing on each lower limb. Using the distribution of the center of pressure of the two feet during standing and squatting, changes in static balance was tested with or without wearing the device. The bracing system and Exotendon stabilize the subject's weak side of the affected limb, allowing the subject to increase weight bearing of the affected leg while maintaining stability, and the difference in the distribution of the center of pressure of the two feet became smaller. A reduction in weight bearing asymmetry after wearing the devices indicates that the Kickstart walking system improves postural control by providing support for the body. It appears that one subject (#16) who was symmetrical in the balance test without the exoskeleton device, after wearing the device became more asymmetrical possibly due to the additional weight of the exoskeleton on the impaired side (Fig. 2b). This may indicate a difficulty in controlling balance with the added weight of the device or a usefulness of the device for weight bearing on the affected side during postural tasks. However, this is one subject and we would need more data to make stronger inferences.

### Spatio-temporal gait measures

Regardless of clinical application and research, walking speed is often an objective assessment tool for functional activities [38]. Decline in walking speed has been recognized as an indicator of underlying dysfunctions that can have serious consequences such as limited mobility, hospitalization, inability to live independently or even death [39–42]. An increase in walking speed is therefore considered to be an indicator of improvement in the quality of life and functional performance in stroke survivors [43]. Therefore, devices that can improve walking speed in stroke survivors are important for rehabilitation. The 10MWT is a test widely used to evaluate walking performance [44–49]. The reduction in time taken to perform the 10MWT while wearing the device showed that the Kickstart walking system was such a device. When stroke survivors wore the Kickstart walking system, the Exotendon stored energy during the stance phase of the gait and released it during the swing phase of the gait. Consequently, paretic propulsion was improved which meant that the more affected side required less propulsive power from its weakened muscles. This led to an improvement in walking performance and reduction in the time taken to perform the 10MWT. For the 10MWT, if we consider an arbitrary threshold of 20 s, then a visual inspection of Fig. 2c shows that most low functioning individuals reduced their time while high functioning individuals were stable and didn’t show much change. This could be due to the device being more useful for more severe cases but may also be due to a ceiling effect in the high functioning individuals.

There was no significant improvement in the TUG test after wearing the Kickstart walking system. The timed-up and go test provides an easy and quick assessment of the stroke survivor’s functional mobility [50]. The results may not be surprising because the TUG test has components of transitioning from sitting to standing and then walking. Although the Kickstart walking system has clear benefits for walking, its usefulness in transitioning from sitting to standing may either be limited or may need more practice to master.

After wearing the Kickstart walking system, there was a reduction in the step time of the affected side and the DST. This happened without a significant reduction in step length. Taken together with the 10MWT results, this means that stroke survivors were able to walk faster without compromising on distance traveled or length of steps taken. Step time in stroke survivors is known to be longer and step length is shorter than controls and these can be improved with training [51]. Assistance from the Kickstart walking system was therefore instrumental in reversing these stroke symptoms. Long duration training with the passive device has therefore strong potential in making these acute observations more permanent. In addition, stroke survivors also demonstrate long durations of DST that can be reduced with training [51]. This is because stroke symptoms like paretic propulsion deficit, gait asymmetry, weight bearing asymmetry, all reduce the amount of time spent on single limb support and increase DST. By providing paretic propulsion assistance, improving weight bearing asymmetry, improving stability, the Kickstart walking system is able to improve walking efficiency and reduce DST [52, 53].

Gait analysis also showed that stroke survivors experienced a significant widening of step width after wearing the Kickstart walking system. This is possibly related to the relatively large training shoe that is part of the passive device. While stroke survivors are known to walk with a wider gait in comparison to healthy controls [54], the reason here is possibly device-related because stroke survivors are also known to walk slowly which is opposite to the exoskeleton-assistance effect in our study.

### Exoskeleton-use feedback questionnaire

One important part of this feasibility study was to determine if stroke survivors felt positive about utilizing the device for improving their functional outcomes. The results were mixed. In general stroke survivors felt that using the exoskeleton improved stability, gait and walking speed which importantly were also reflected in our biomechanical measures. In addition, stroke survivors agreed that they had confidence in the device and were willing to use it. These findings were similar to another study for the ReWalk exoskeleton device [55]. Interestingly that study also scored average for comfort and ease of wearing and adjusting the device. It is important to note that the questionnaire was done after early exposure to the device and as the study progressed, the study team had a better idea of how to best fit the device according to individual stroke deficits. This is also the reason the first 10 subjects did not have a specific tension in their Exotendon (Table 2).

This study had certain limitations. Subjects could use the device for a maximum of only 5 days. Although a pre-post study would have been ideal, the study aimed to test the feasibility of walking with an exoskeleton device in a sample of stroke survivors after familiarizing them with the device over the 5-day period. The effects over the 5-day period also include the effects of the inpatient rehabilitation that the subjects received. However, even after this short exposure, significant differences were noted that we anticipate will be consolidated when our longer duration study is completed in a larger sample of stroke survivors that would include a control group receiving only inpatient rehabilitation. Several other measures like cadence, limp index and step length did not show differences when tested with or without the exoskeleton device. This is not necessarily a negative outcome. We have to remember that this was not a pre-post study, rather a feasibility study and so we were also exploring if the device hampered gait outcomes in a sample of stroke survivors. We found that this was not the case. This gives us confidence to proceed to a longer duration study with more comprehensive pre- and post-testing in a larger sample of stroke survivors. In addition, we will also consider feedback from physical therapists regarding device fitting and ease of use in our future studies. Finally, participants were allowed to use other assistive devices when using the exoskeleton. Therefore, not all subjects used the device during exoskeleton-assisted gait training. The impact of exoskeleton-assisted gait training on assistive device usage would be interesting to investigate in our longer duration study.

Although this study does not provide direct evidence of feasibility for longer periods of treatment however, we would like to pursue this line of thought for a few reasons. First, we did not have an adverse event during the study. Second, this device has been safely used clinically for a number of years now in patients with neurological deficits. Most of this data is clinical and only a case series has been published [35]. The paper describes a spinal cord injury patient who used the device for 8 months, and two stroke survivors one of whom used the device for 2 months and the other for 12 months. The stroke survivors also used the device at home without supervision. The 6-min walk test (6MWT) provided important evidence as to the usefulness of the exoskeleton device. In the 3 cases, the distance for the 6MWT improved from 25 to 125 m, 123 to 224 m and 120 to 226 m.

## Conclusion

In this feasibility study, our aim was to test the Kickstart^®^ Walk Assist system which is a passive lower limb exoskeleton device, in a sample of stroke survivors. The study specifically targeted balance, gait and walking efficiency of the study participants. In addition, the participants were also surveyed for determining their perceptions of functional improvement and comfort issues encountered when using the device. Study participants were tested for several measures with/without the device after experiencing walking with the exoskeleton over a duration of 5 days. Significant reductions were determined in the 10MWT, weight bearing asymmetry, step time, and DST. In addition, no adverse events were noted in the participants. These findings show that the exoskeletal device has short-term feasibility and therefore, using the device for longer periods would be feasible for stroke survivors.

## Supplementary information

**Additional file 1.** CONSORT flow diagram.

## Data Availability

Not applicable.

## Abbreviations

10MWT: 10-meter walking test
TUG: Timed up and go
DST: Double support time
