# Supplementary material for: The Kickstart Walk Assist System for improving balance and walking function in stroke survivors: a feasibility study
Source: J Neuroeng Rehabil. 2021 Feb 24;18:42. doi: 10.1186/s12984-020-00795-y (PMC7905648; doi:10.1186/s12984-020-00795-y)
Supplement: Supplementary file 1 — Additional file 1. CONSORT flow diagram. [file 12984_2020_795_MOESM1_ESM.docx]

Assessed for eligibility (n=35)

Excluded (n=0)

Walking efficiency (n=30)

Balance Performance (n=30)

Gait analysis (n=30)

- Excluded from analysis (n=5)
  - failed to complete one or more evaluation tasks

Lost to follow-up (give reasons) (n=0)

Discontinued intervention (give reasons) (n=0)

Allocated to intervention (n=35)

- Attended at least one training session of duration 20 minutes per day for 5 days with the exoskeleton device

Allocation

Assessment

Follow-Up

Enrollment

＊Modified for non-randomized trial design
